# The Effect of the Physical and Mental Exercises During Hemodialysis on Fatigue: A Controlled Clinical Trial

**DOI:** 10.5812/numonthly.14686

**Published:** 2014-07-05

**Authors:** Zeynab Motedayen, Batool Nehrir, Ali Tayebi, Abbas Ebadi, Behzad Einollahi

**Affiliations:** 1 Nephrology and Urology Research Center, Baqiyatallah University of Medical Sciences, Tehran, IR Iran; 2 School of Nursing, Baqiyatallah University of Medical Sciences, Tehran, IR Iran

**Keywords:** Planning Techniques, Exercise, Renal Dialysis, Fatigue

## Abstract

**Background:**

Despite regular treatment by hemodialysis, patients on hemodialysis are affected by uremic syndrome, which is marked by fatigue. Fatigue is supposed as the most common and the most severe symptom ever reported by patients with chronic kidney disease.

**Objectives:**

This controlled study aimed to evaluate the effect of intradialytic physical and mental exercises on fatigue in patients on hemodialysis.

**Patients and Methods:**

A total of 66 patients on long-term hemodialysis were selected via purposive sampling and were assigned to two groups, namely, control and experimental groups. The experimental group participated in a intradialytic training program twice a week for two months. The program was designed by a senior expert in physical education. Their fatigue was measured via a fatigue severity scale questionnaire before as well as one and two months after the intervention in both groups.

**Results:**

The mean of the fatigue score within the research units was 42.37. Overall, 42.2% and 56.1% of the participants experienced medium and severe fatigue, respectively. The scores of fatigue decreased significantly from the beginning through two months after intervention in the experiment group.

**Conclusions:**

With respect to the findings of the study, this method of treatment is recommended due to being cost efficient, easy, applicable, and flexible for alleviating the effect of fatigue on the personal, psychological, and social aspects of life quality in patients on hemodialysis.

## 1. Background

In order to survive, patients with end-stage renal disease need renal replacement therapies such as hemodialysis, peritoneal dialysis, or kidney transplantation (1-4). Despite regular hemodialysis, the patients are still affected by a symptomatology, i.e. uremic syndrome, with fatigue being one of the most common symptoms (4). Subsequent to the uremic syndrome, the patients’ physical work capacity decreases by 50% in comparison with the healthy individuals (4). There is a significant correlation between the reduction of physical activity and increased depression or fatigue. Due to decreased activity, the individual’s strength decreases, which intensifies the depression and fatigue (5, 6).

In general, fatigue has been described as weakness, feeling of exhaustion, and lack of energy (7). Fatigue not only affects the patients’ everyday life but also causes impaired daily self-care, psychologic status, and the quality of life (1, 6, 8). About 94% of patients on hemodialysis tend to undergo more sessions of dialysis if it would increase their energy level (9, 10); however, few studies have been conducted in this regard (11, 12). Fatigue, with the prevalence of 60% to 97%, has been proposed as the most severe symptom ever reported among patients with chronic kidney disease (CKD) (7, 8, 10, 13, 14). The study by Jhamb et al. indicated that patients with end-stage renal disease experience extreme levels of fatigue (9). Nonetheless, it is less recognized or treated due to its subjective nature (1, 15).

The treatment method for fatigue is classified into two groups, i.e. pharmacological and nonpharmacological (16). The former method involves L-carnitine-, vitamin C, and prescription of erythropoietin and other medications to control anemia (7, 16). The latter method involves exercise, yoga, relaxation, acupressure, acupuncture, electric stimulation, and dialysis (7, 12, 16, 17). Kao et al. showed that exercise might be helpful in alleviating the depression and fatigue among patients on dialysis (6). Exercise was performed in such various ways as aerobic, aerobic-strength, endurance, and resistance exercises as well as leg ergometric exercises and progressive muscle relaxation (18-21). Some studies suggested that imagery techniques and relaxation could be successfully employed to improve the adjustment condition of patients on dialysis (22).

## 2. Objectives

To our knowledge, there was no study concerning the mental and physical exercise during dialysis. According to the high prevalence of CKD, low level of activity, and higher prevalence of fatigue among the patients on dialysis, the current study aimed to investigate the effect of intradialytic physical and mental exercises on fatigue among these patients.

## 3. Patients and Methods

### 3.1. Patients

In this controlled clinical trial, the patients on hemodialysis admitted to Baqiyatallah Hospital and Labbafinejad Hospital, Tehran, Iran, were recruited to the experimental and control groups, respectively. The sampling was purposive and patients undergoing hemodialysis thrice a week with at least three months since the start of hemodialysis who were capable of learning during the exercises were included. Patients with the following conditions were excluded: participating in the regular exercise program in the preceding six months, medical prohibition from the exercise, history of ischemic heart disease, third degree congestive heart failure, unstable angina, or kidney transplant, high blood pressure (≥ 180/110 mmHg), low blood pressure (≤ 90 mmHg), and reluctance to continue participating in the exercises.

### 3.2. Data collection

Fatigue severity scale (FSS) questionnaire and a demographic characteristics form including age, sex, marital status, duration of hemodialysis per month, education, employment, the effect of treatment on the job status, and the cause of renal failure were used for data collection. The validity and reliability of FSS was previously proved in a study by Zakerimoghadam (23). FSS includes nine questions rated from one to seven (24). The total score indicating the fatigue measure was calculated by dividing the sum of all scores by nine. Score one indicated lack of fatigue, two to four indicated medium fatigue, and five and above meant severe fatigue (25). The FSS questionnaire was completed by the subjects prior to the study and at the end of the first and the second months.

### 3.3. Study Design

Prior to the intervention, patients were assured that their information would be kept totally confidential and their anonymity was ensured. They were informed that whenever they wished they could leave the study. Then a written informed consent was obtained from each patient. Confidentiality and anonymity was guaranteed with respect to the knowledge of the staff about the patients’ information and findings.

Each patient was initially questioned about their kinetic limitations and comorbid diseases in order to design an exercise program corresponding to their individual capabilities. The exercises were instructed by a senior expert in physical education during the dialysis, half an hour after the patients were connected to the dialysis machine. At the beginning of each session, the trainer began taking about the system of the universe, the infinite power of Almighty God, positive thinking about the self, and avoiding the vices, fouls, and disturbing thoughts. Then the patients were encouraged to do stretching and flexibility movements in the muscles of their neck, arm, abdominal, thigh, and shin as well as in their shoulder, elbow, wrist, spinal, knee, and ankle joints. For the limb with fistula, the patient was instructed to close their eyes and imagine moving it. During the exercise intervals, the patients were required to take a deep breath or do diaphragm breathing. Ultimately, they were taught to do relaxation exercises under the trainer’s guidance while a soft music without lyrics was being played. In the next sessions the exercises were extended in terms of strength and repetition with respect to the subjects’ capabilities. The exercises would be stopped in cases of witnessing fainting, dyspnea, nausea, vomiting, chest pain, joint pain, or muscle pain. They were continued twice a week for almost two months with a maximum duration of 20 minutes. 

### 3.4. Statistical Analysis

For statistical analysis, SPSS (version 18, SPSS Inc., Chicago, IL, USA) was employed and Chi square, Fisher’s exact test, Mann-Whitney U test, one-way ANOVA, and Friedman test were used for variables without normal distribution and t test was used for quantitative variables with normal distribution.

## 4. Results

Initially, 75 patients were assigned to the experimental and control groups; nine patients were excluded from the study because of death, transplantation, transportation from the health center, or refusing to do the exercises regularly due to fatigue, boredom, and sleeplessness on the night before dialysis. Therefore, the findings of the study were extracted from the information of two 33-patient groups. 

Prior to any parametric data processing, we verified normality of sample distribution via Kolmogorov-Smirnoff test and homogeneity of variance through Levene’s test. Homogeneity of both groups in terms of the qualitative and quantitative variables was established via Chi square and independent samples t test, respectively (Table 1). There were 38 males (57.6%) and 50 married (75.8%) participants amongst all the patients. the mean age of participants was 56.75 ± 11.91 years. Diabetes was the most common cause of renal failure among the subjects (28.8%).

**Figure 1.**
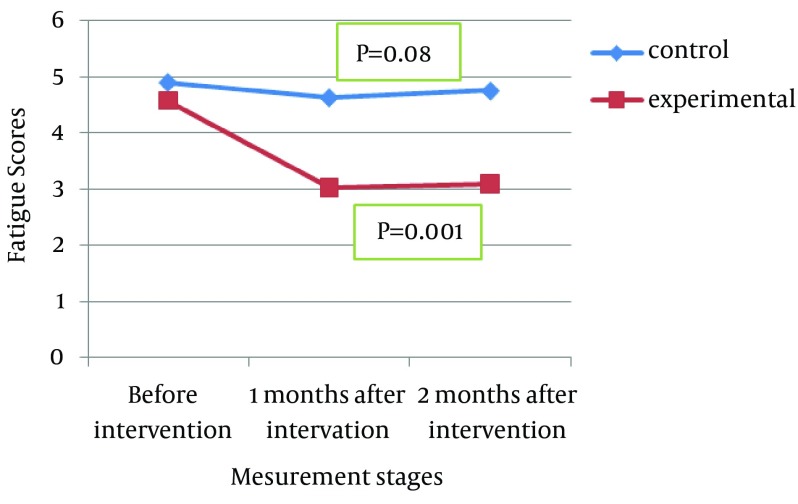
Trend of Fatigue Scores Changes in Control and Experimental Groups

Medium and sever fatigue were detected in 42.4% and 56.1% of the subjects, respectively. Although there was a significant difference between the fatigue scores before and after the intervention in the experimental group, such a difference was not detected in the control group (Figure 1). The findings concerning the comparison of frequency and mean of the fatigue scores before and after the intervention in the study groups are shown in Table 2.

**Table 1.** Distribution of Study Subjects According to Demographic Characteristics ^a^

Variables	Control	Experimental	P Value
**Sex**			0.13
Female	17 (51.5)	11 (33.3)	
Male	16 (48.5)	22 (66.7)	
**Age, y**			0.62
30-55	17 (51.5)	15 (45.5)	
56-75	16 (48.5)	18 (54.5)	
**Employment**			0.12
Unemployed	24 (72.7)	29 (87.9)	
Employed	9 (27.3)	4 (12.1)	
**Education**			0.15
Illiterate	3 (9.1)	1 (3)	
Below Diploma	13 (39.4)	11 (33.3)	
Diploma	6 (18.2)	14 (42.5)	
Academic	11 (33.3)	7 (21.2)	
**Marital status**			0.22
Single	5 (15.2)	1 (3)	
Married	23 (69.6)	27 (81.8)	
Widow/Widower/Divorced	5 (15.2)	5 (15.2)	
**Dialysis duration, mo**			0.15
< 24	14 (42.4)	15 (45.5)	
24-120	6 (18.2)	11 (33.3)	
> 121	13 (39.4)	7 (21.2)	
**Causes of Renal Failure**			0.08
Blood Pressure	4 (12.1)	3 (9.1)	
Diabetes	4 (12.1)	15 (45.4)	
Vascular Problems	6 (18.2)	3 (9.1)	
Infection	7 (21.2)	3 (9.1)	
Unknown	5 (15.2)	3 (9.1)	
Other Causes	7 (21.2)	6 (18.2)	
**Hemoglobin**			0.32
≤ 11	20 (60.6)	16 (48.5)	
> 11	13 (39.4)	17 (51.5)	

^a^Data are presented as No. (%).

**Table 2.** Fatigue Severity in Study Groups According to the Time of Measurement ^a^

Group	No Fatigue	Medium	Severe	Friedman Test	
**Control**				χ ^2^ = 4.93 P = 0.08	χ ^2^ = 36.77 P = 0.001
Before Study	0 1 (3) 0	13 (39.4)	20 (60.6)		
1 Month Later	-	14 (42.5)	18 (54.5)		
2 Months Later	-	16 (48.5)	17 (51.5)		
**Experimental**				χ ^2^ = 35.35 P = 0.001	
Before Study	1 (3)	15 (45.5)	17 (51.5)		
1 Month Later	6 (18.1)	22 (66.7)	5 (15.2)		
2 Months Later	4 (12.1)	24 (72.7)	5 (15.2)		

^a^ Data are presented as No. (%).

No significant difference was detected between variables such as sex, education, marital status, cause of renal failure, and the fatigue (Table 3); however, a significant difference was observed between fatigue and duration of dialysis as well as employment. Moreover, by employing the Spearman test, a significant correlation between the fatigue and the variable of negative effect of dialysis on employment was observed (P = 0.001, r = 0.41). The age and the total score of fatigue had a significant positive correlation (P = 0.006, r = 0.33).

**Table 3.** Fatigue Scores Regarding Demographic Characteristics

Variables	Mean ± SD	P Value
**Sex**		0.49
Male	4.57 ± 1.75	
Female	4.86 ± 1.75	
**Education**		0.30
Illiterate	5.5 ± 1.29	
Below Diploma	5.08 ± 1.71	
Diploma	4.70 ± 1.71	
Academic	4.16 ± 1.85	
**Marital Status**		0.27
Single	3.66 ± 1.86	
Married	4.88 ± 1.62	
Widow	4.70 ± 2.21	
**Causes Of Renal Failure**		0.66
Blood Pressure	3.85 ± 1.34	
Diabetes	5.10 ± 1.79	
Vascular Problems	4.77 ± 1.85	
Infection	4.40 ± 1.64	
Unknown	4.62 ± 1.40	
Other Causes	5 ± 2.12	
**Employment**		0.005
Employed	3.53 ± 1.26	
Unemployed	5.03 ± 1.73	
**Dialysis Duration, Months**		0.04
< 24	5.26 ± 1.48	
≥ 24	4.40 ± 1.83	
**Hemoglobin**		0.42
≤ 11	4.58 ± 1.76	
> 11	4.93 ± 1.74	

## 5. Discussion

Fatigue is a frequent symptom shared by all patients on long-term dialysis (15). Joshwa et al. reported fatigue in 75% of the patients on hemodialysis (24); in the current study, 65.2% of the patients had experienced fatigue.

In this study, there was a significant difference between the fatigue scores in the experimental group before and after the intervention, which was due to intradialytic mental and physical exercises for two months. The study conducted by Riahi et al. demonstrated significant reduction in the fatigue level after five months of intradialytic use of leg ergometric exercises, which was in line with the findings of the current study (26). In study of Yurtkuran et al., a significant improvement was observed in the patients’ fatigue after 12 weeks of yoga exercises (27). This is in contrary to the fact that the study had cost the patients their extra time by being performed in a time other than the dialysis hours. Chang et al. reported a significant reduction in the fatigue level after eight consecutive weeks of intradialytic use of leg ergometric exercises (18), while the current study did not require using any special tool and was easily conducted in an occupied space of the hospital ward.

In our study, no significant correlation was observed between patients’ sex and fatigue. There is evidence indicating that fatigue in women happens with greater sequence and severity in comparison with men; however, it was not confirmed by all the studies conducted so far (16). The study by Chang demonstrated no significant difference between sex and the fatigue or physical activity (18), while in the study by Liu the fatigue was greater in women than men (28). 

Age served as a variable predicting the fatigue in our study. With age, the total score for fatigue increased; however, Chang et al study (18) had considered physiological changes due to the effect of a chronic disease as the cause of increase in fatigue with age.

Although the spouses of the patients on hemodialysis played a supporting role in reducing the stress, adapting to the disease, following the treatment, and reducing the weakness, the findings of some studies were not indicative of any significant difference between fatigue severity and marital status (29), which were in agreement with the findings of the current study.

Compared to the employed individuals, the unemployed ones exhibited greater fatigue in our study, which was confirmatory to the findings by Liu (28). Increase in motivation as well as social interactions, decrease in the fatigue occurrence, lack of concern for economic problems, and increase in the level of activity might justify the reduction in the fatigue among the employed individuals. On the contrary, staying at home led to a reduction in the physical activity and losing the colleagues’ social support. Thus, the unemployed patients might report a higher level of fatigue.

Having the benefit of higher education allowed the patients to employ fatigue-reducing solutions. The findings of a previous study indicated that as the level of education increased, the subjects’ fatigue decreased (29). In the current study, the illiterate and the university educated subjects reported the highest and the lowest level of fatigue, respectively; however, the difference was not statistically significant.

The present study demonstrated that patients with less than 24 months since their dialysis exhibited greater fatigue, while according to Liu’s study, no correlation existed between the duration of dialysis and any of the fatigue dimensions (28). Interestingly, the study by Letchmi et al. pointed toward a significant correlation between fatigue and duration of dialysis (30), which was in accordance with the findings of the current study. Therefore, the negative correlation between the duration of dialysis and fatigue might be indicative of the fact that patients undergoing dialysis for less than two years were not yet capable of developing coping behavior in line with the treatment.

Diabetes is identified as the single most common cause of gradual loss of kidney function (31). Based on the findings of the present study, diabetes was the most common cause of renal failure; however, it was not statistically significant. Furthermore, individuals with diabetes exhibited more fatigue possibly because of the disease process and its ensuing consequences. The study conducted by Raimann et al. demonstrated that postdialysis fatigue is more severe in patients with diabetes than other patients (32). One of the reasons of fatigue in diabetes is fluctuations in blood glucose levels. Fatigue may also be correlated to psychological factors, such as depression or emotional problems related to the diagnosis or to the intensity of diabetes self-management regimens. Fatigue may also be related to such lifestyle methods as lack of physical activity or being overweight (33).

In Conclusion, the positive effects of exercise, regarding performance time and method, on improvement of the quality of life, reduction of cardiovascular complications, mortality rate, depression, sleep, and fatigue has been documented clearly. The present study showed that simple intradialytic physical and mental exercises programs, with no need to expensive and highly-morbid procedures, can decrease the fatigue in patients on hemodialysis. Therefore, this therapeutic method is recommended for this group of patients.
